# DCIS and axillary nodal evaluation: compliance with national guidelines

**DOI:** 10.1186/s12893-017-0210-5

**Published:** 2017-02-07

**Authors:** Katrina B. Mitchell, Heather Lin, Yu Shen, Alfred Colfry, Henry Kuerer, Simona F. Shaitelman, Gildy V. Babiera, Isabelle Bedrosian

**Affiliations:** 1 Department of Breast Surgical Oncology, University of Texas, MD Anderson Cancer Center, Houston, TX 77030 USA; 2 Department of Biostatistics, University of Texas, MD Anderson Cancer Center, Houston, TX USA; 3 Department of Radiation Oncology, University of Texas, MD Anderson Cancer Center, Houston, TX USA

**Keywords:** DCIS, Surgery, Axillary staging, Cancer care guidelines

## Abstract

**Background:**

The National Comprehensive Cancer Network (NCCN) and the American Society of Clinical Oncology (ASCO) provide guidelines regarding axillary nodal evaluation in ductal carcinoma *in situ* (DCIS), but data regarding national compliance with these guidelines remains incomplete.

**Methods:**

We conducted a retrospective review of the National Cancer Data Base (NCDB) analyzing all surgical approaches to axillary evaluation in patients with DCIS. Logistic regression analysis was used to assess the multivariate relationship between patient demographics, clinical characteristics, and probability of axillary evaluation.

**Results:**

We identified 88,083 patients diagnosed with DCIS between 1998 and 2011; 31,912 (37%) underwent total mastectomy (TM) and 55,349 (63%) had breast conserving therapy (BCT). Axillary evaluation increased from 44.4% in 1998 to 63.3% in 2011. In TM patients, axillary evaluation increased from 74.3% in 1998 to 93.4% in 2011. This correlated with an increase in sentinel lymph node biopsy (SLNB) from 24.3 to 77.1%, while ALND decreased from 50.0 to 16.3% (*p* <0.01). In BCT patients, evaluation increased from 20.1 to 43.9%; SLNB increased from 7.2 to 39.4% and ALND decreased from 12.9 to 4.5%. Factors associated with axillary nodal evaluation in BCT patients included practice type and facility location. Among TM patients, use of axillary lymph node dissection (ALND) for axillary staging was associated with earlier year of diagnosis, black race, and older age, as well as community practice setting and practice location in the Southern US.

**Conclusions:**

Compliance with national guidelines regarding axillary evaluation in DCIS remains varied. Practice type and location-based differences suggest opportunities for education regarding the appropriate use of axillary nodal evaluation in patients with DCIS.

## Background

Ductal carcinoma *in situ* (DCIS) currently represents 20% of breast cancers, affecting approximately 65,000 women per year [1, 2]. Since the early 1970s, the incidence of DCIS has increased from 1.8 per 100,000 women to 32.5 per 100,000 women in the mid-2000s [3]. This represents a fivefold increase in diagnoses over time, and largely has resulted from widespread adoption of screening mammography: more than 80% of lesions are detected through this modality [4]. Though DCIS carries a low cumulative 20-year breast cancer specific mortality rate of 3.3%, its diagnosis nevertheless may trigger potentially aggressive interventions with associated complications [5]. In particular, axillary interventions, even limited intervention through use of sentinel node biopsy, carry risk of long term sequelae such as chronic pain, decreased strength, edema, and sensory disorder from sentinel lymph node biopsy [6].

Though DCIS treatment traditionally has involved a combination of radiation, endocrine therapy, and surgery, attention is increasingly being directed towards analyzing the appropriateness of surgical interventions. In particular, the rate and appropriateness of axillary lymph node evaluation in surgical procedures for DCIS recently has been investigated [7–11]. Many of these studies acknowledged that while DCIS represents in-situ disease and the vast majority of patients do not require axillary evaluation, selected cases may warrant the procedure.

Guidelines set forth by the National Comprehensive Cancer Network (NCCN) do not recommend axillary lymph node evaluation for patients undergoing breast conserving therapy (BCT); it advises that mastectomy (TM) patients may or may not undergo sentinel node biopsy (SLNB). The NCCN further states that axillary lymph node dissection (ALND) should not be performed for DCIS in the absence of invasive cancer or proven axillary disease, but does note that invasive cancer may be found at the time of surgery in a small percentage of patients. Because of this, the NCCN recommends that SLNB should be “strongly considered” with mastectomy or “excision in an anatomic location compromising the performance of a future sentinel lymph node procedure.” [12].

Similarly, in its 2014 summary of guidelines addressing sentinel lymph node biopsy for patients with early-stage breast cancer, ASCO reaffirmed that clinicians may offer SLNB to patients undergoing a mastectomy (www.asco.org/guidelines/snbbreast). It does not recommend SLNB for BCT unless the tumor is in a location that may preclude future node sampling due to lymphatic disruption during the index procedure; DCIS diagnosed as a mass on imaging or clinical exam; or, large volume DCIS [13].

Overall, the majority of DCIS lesions would not fall within these guidelines and as such axillary evaluation would be considered over treatment. The purpose of this study was to evaluate adherence with these national guidelines and identify areas in which clinical care and education may be focused to improve compliance and reduce unnecessary axillary nodal evaluation. This will prevent both under and overtreatment of DCIS, eliminating unnecessary patient morbidity and reducing health care costs.

## Methods

### Data source

The National Cancer Data Base (NCDB) represents a collaborative repository of patient data drawn from the American Cancer Society (ACS) and the Commission on Cancer (COC) of the American College of Surgeons (https://www.facs.org/quality-programs/cancer/ncdb) Since 1989, the NCDB has functioned as a nationwide oncology outcomes database for more than 1,500 COC-accredited cancer programs in the United States (US) and Puerto Rico, and contains approximately 29 million total records. Seventy percent of all newly-diagnosed cancers are captured at the institutional level and reported to the NCDB. Since patient level identifiers are not available to NCDB users, this study was exempted from IRB review and approval.

### Selection of analytic cohort

From the total 2,720,347 patients in the dataset, 88,083 female patients who met the following DCIS criteria were included in the final analytic cohort: 1) had a histology code of 8010, 8020, 8022, 8201, 8230, 8500, 8501, 8503, 8514, 8521, 8522, 8523, 2) were clinically designated as TisN0M0, 3) were pathologically confirmed TisN0M0, 4) had no history of other prior cancers and 5) did not receive chemotherapy. Patients whose insurance status was unknown or who were treated at “other specified types of cancer programs” were excluded from the analysis.

### Statistical analysis

SLNB was defined as 1–4 lymph nodes removed, while ALND was defined as ≥5 lymph nodes removed. Univariate analysis was performed to evaluate the association between each variable and axillary evaluation, using chi-square tests for categorical variables and *t*-test/ANOVA or the counterparts of the non-parametric approaches (Wilcoxon rank-sum or Kruskal-Wallis) for continuous variables [14]. Logistic regression analysis [15] was used to assess the multivariate relationship between patient demographic, clinical characteristics and the probability of axillary evaluation. Age was categorized into quartiles. SAS version 9.2 and S-Plus version 8.04 was used to carry out the computations for all analyses.

## Results

**Fig. 1:**
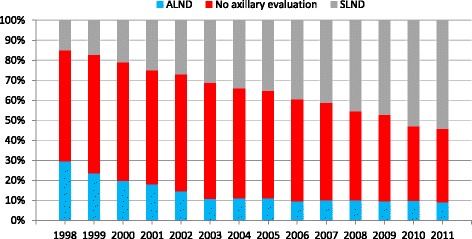
Trend in type of axillary evaluation over time among all patients

Of the 88,083 patients diagnosed with DCIS from 1998 to 2011 who met inclusion criteria for our analysis, 31,912 of these patients (37%) underwent total mastectomy (TM), and 55,349 patients (63%) underwent BCT. For the entire cohort of patients evaluated, axillary lymph node evaluation (either ALND or SLNB) increased from 44.4% in 1998 to 63.3% in 2011, with an increasing proportion of SLNB utilized over time: ALND decreased from 29.5 to 9.1%, while SLNB increased from 14.9 to 54.2% (*p* <0.01). Figure 1 illustrates this trend.

**Fig. 2:**
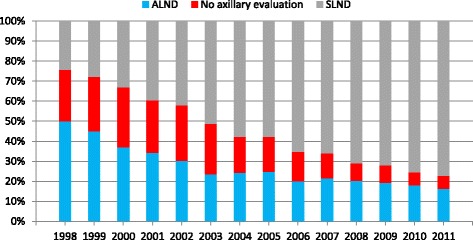
Trend in axillary evaluation over time among patients who underwent mastectomy

Among patients who underwent TM, a similar trend of overall increased axillary evaluation from 74.3% in 1998 to 93.4% in 2011 was demonstrated. As with the entire cohort, this correlated with a concomitant increased utilization of SLNB: ALND decreased from 50.0 to 16.3%, while SLNB increased from 24.3 to 77.1% (*p* <0.01). Figure 2 illustrates this trend.

In the TM cohort, younger age was significantly associated with axillary evaluation: 88.5% of 18–46 years-old patients underwent an axillary procedure, compared with 82.1% of those older than 65 (*p* <0.01) (Table 1). Treatment at an academic/research program also was significantly associated with axillary evaluation, with 88.1% of patients undergoing a procedure in those institutions, versus 86% at a comprehensive community cancer program and 81% at a community cancer program (*p* <0.01). Although facility location in the South had a higher rate of axillary evaluation as compared with the other geographic areas (*p* = 0.02), absolute differences between geographic locations were relatively small (2%). Overlapping lesions underwent the highest rate of axillary evaluation versus the lowest at the nipple/areola (88 vs. 79.1%) (*p* <0.01). Tumors <1 cm had lower axillary evaluation rates compared with tumors >5 cm (83.5 vs. 92.8%, *p* <0.01).

**Table 1:** Univariate analysis of axillary evaluation and patient characteristics among those who underwent mastectomy

		Mastectomy Cohort (*n* = 31,912)			Breast Conservation Cohort (*n* = 55,349)		
Variable		Yes	No	*p*-Value	Yes	No	*p*-Value
Year of Diagnosis	1998	828(74.4%)	285(25.6%)	<0.01	265(20.1%)	1051(79.9%)	<0.01
	1999	821(72.8%)	306(27.2%)		303(18.8%)	1311(81.2%)	
	2000	838(70.1%)	358(29.9%)		369(21.1%)	1378(78.9%)	
	2001	896(73.7%)	319(26.3%)		437(23.6%)	1412(76.4%)	
	2002	895(72.4%)	342(27.6%)		503(24.1%)	1582(75.9%)	
	2003	1146(74.9%)	385(25.1%)		819(26.5%)	2272(73.5%)	
	2004	1203(82.1%)	262(17.9%)		895(28.5%)	2250(71.5%)	
	2005	1345(82.5%)	286(17.5%)		1008(29.9%)	2365(70.1%)	
	2006	1544(85.1%)	270(14.9%)		1216(32.3%)	2553(67.7%)	
	2007	1939(87.5%)	278(12.5%)		1539(34.1%)	2979(65.9%)	
	2008	3471(91.3%)	329(8.7%)		2611(36.9%)	4457(63.1%)	
	2009	4142(91.3%)	397(8.7%)		3041(37.9%)	4981(62.1%)	
	2010	4151(93.5%)	287(6.5%)		2904(43%)	3851(57%)	
	2011	4286(93.4%)	303(6.6%)		3069(43.9%)	3928(56.1%)	
Race	Black	3144(86.5%)	491(13.5%)	0.52	2087(34.1%)	4039(65.9%)	0.02
	White	22682(86.2%)	3638(13.8%)		15954(34.5%)	30246(65.5%)	
	Other	1335(85.3%)	230(14.7%)		761(31.9%)	1628(68.1%)	
Age	18–46	7089(88.5%)	918(11.5%)	<0.01	5023(34.8%)	9392(65.2%)	<0.01
	47–55	7440(87.2%)	1096(12.8%)		5137(34.9%)	9584(65.1%)	
	56–65	6549(86.8%)	992(13.2%)		4743(35.6%)	8594(64.4%)	
	>65	6427(82.1%)	1401(17.9%)		4076(31.7%)	8800(68.3%)	
Facility Type	Community Cancer Program	2175(81%)	511(19%)	<0.01	2133(36.4%)	3731(63.6%)	<0.01
	Comprehensive Community Cancer Program	16873(86%)	2757(14%)		12474(36.6%)	21632(63.4%)	
	Academic/Research Program	8457(88.1%)	1139(11.9%)		4372(28.4%)	11007(71.6%)	
Facility Location	Midwest	6742(86.2%)	1079(13.8%)	<0.01	4169(31.4%)	9128(68.6%)	<0.01
	Northeast	5267(86.7%)	810(13.3%)		3612(27.9%)	9319(72.1%)	
	South	10642(86.8%)	1623(13.2%)		7738(41.2%)	11023(58.8%)	
	West	4854(84.4%)	895(15.6%)		3460(33.4%)	6900(66.6%)	
Primary Site	Nipple areolar	223(79.1%)	59(20.9%)	<0.01	94(26.7%)	258(73.3%)	<0.01
	Central	1766(85.1%)	310(14.9%)		1047(30.7%)	2368(69.3%)	
	Upper inner	1883(86.5%)	294(13.5%)		1629(34.1%)	3153(65.9%)	
	Lower inner	1672(87.3%)	244(12.7%)		1123(32.9%)	2288(67.1%)	
	Upper outer	7503(86.4%)	1185(13.6%)		7413(36.7%)	12801(63.3%)	
	Lower outer	1861(86.8%)	284(13.2%)		1240(33.9%)	2418(66.1%)	
	Axillary tail	31(86.1%)	5(13.9%)		51(48.1%)	55(51.9%)	
	Overlapping lesion	6113(88%)	835(12%)		3723(34.2%)	7175(65.8%)	
	NOS	6453(84.4%)	1191(15.6%)		2659(31.2%)	5854(68.8%)	
Tumor Size	≤1 cm	4921(83.5%)	970(16.5%)	<0.01	5605(30.3%)	12878(69.7%)	<0.01
	>1 cm, ≤2 cm	5846(88.7%)	745(11.3%)		4903(40.7%)	7139(59.3%)	
	>2 cm, ≤3 cm	1762(90%)	195(10%)		940(43.9%)	1199(56.1%)	
	>3 cm, ≤4 cm	1376(89.6%)	159(10.4%)		647(45.3%)	781(54.7%)	
	>4 cm, ≤5 cm	1178(91.7%)	106(8.3%)		363(45.1%)	441(54.9%)	
	>5 cm	2526(92.8%)	196(7.2%)		535(45.2%)	648(54.8%)	

The multivariate analysis of the TM cohort (Table 2) was similar to the results of the univariate analysis. When compared with academic/research programs, community cancer programs as well as comprehensive community cancer programs were less likely to evaluate the axilla (OR 0.63 {95% CI 0.54–0.75}) and (OR 0.84 {95% CI 0.76–0.93}), respectively. Tumors in the nipple/areolar region remained least likely to receive axillary evaluation compared with overlapping tumors (OR 0.57 {95% CI 0.38–0.88}). Smaller tumors (<1 cm) were associated with lower axillary sampling compared with larger tumors (>5 cm) (OR 0.44 {95% CI 0.37–0.52}). Compared with patients diagnosed with DCIS in 2011, those diagnosed in 2000 were the least likely to undergo axillary evaluation (OR 0.15 {0.12–0.20}).

**Table 2:** Multivariate analysis of axillary evaluation and patients’ characteristics among those who underwent mastectomy

Variable		Odds Ratio	Lower CL	Upper CL	*p*-Value
Age	Per year increase	0.99	0.98	0.99	<0.01
Facility Type (ref = Academic/Research Program)	Community Cancer Program	0.63	0.54	0.75	<0.01
	Comprehensive Community Cancer Program	0.84	0.76	0.93	
Primary Site (ref = Overlapping lesion)	Nipple areolar	0.57	0.38	0.88	0.01
	Central	0.82	0.68	0.99	
	Upper inner	0.91	0.76	1.11	
	Lower inner	1.03	0.83	1.26	
	Upper outer	1.00	0.87	1.13	
	Lower outer	0.88	0.72	1.06	
	Axillary tail	2.11	0.47	9.36	
	NOS	0.83	0.72	0.95	
Tumor Size (ref= > 5 cm)	≤1 cm	0.44	0.37	0.52	
	>1 cm, ≤2 cm	0.68	0.58	0.81	
	>2 cm, ≤3 cm	0.74	0.60	0.92	
	>3 cm, ≤4 cm	0.72	0.57	0.90	
	>4 cm, ≤5 cm	0.93	0.72	1.20	
Year of Diagnosis (ref = 2011)	1998	0.19	0.15	0.24	<0.01
	1999	0.19	0.15	0.25	
	2000	0.15	0.12	0.20	
	2001	0.21	0.17	0.27	
	2002	0.18	0.14	0.22	
	2003	0.21	0.17	0.26	
	2004	0.33	0.26	0.42	
	2005	0.35	0.28	0.44	
	2006	0.40	0.32	0.49	
	2007	0.53	0.43	0.66	
	2008	0.76	0.62	0.92	
	2009	0.72	0.60	0.88	
	2010	1.06	0.86	1.29	

To better understand how the patient demographic and provider characteristics influence the use of SLNB versus ALND in the TM cohort, a sub-group analysis was conducted (Table 3). The results showed that year of diagnosis was significantly associated with ALND utilization, with the highest rate in 1998 (67.3%) and lowest in 2011 (17.4%) (*p* = <0.01). Black race was significantly associated with ALND, as 35.1% of black patients underwent ALND versus 26.4% of white patients and 21% of women of other races (*p* <0.01). In contrast to the entire TM cohort, in which younger women were more likely to undergo axillary evaluation, age >65 was significantly associated with the more invasive ALND procedure (32.6 vs 25.2% for 18–46 years, *p* <0.01) compared to SLNB. Facility location in the South was significantly associated with ALND compared to facilities in the West, where the ALND rate was lowest (29.8 vs 23.7%, *p* <0.01), as were community (32.6%) versus academic/research programs (26.2%) (*p* <0.01). In terms of tumor characteristics, locations in the nipple/areola were significantly associated with ALND (37.2%, *p* <0.01), as were those >5 cm in size (27.4 vs 25.2% for tumor <1 cm, *p* <0.01).

**Table 3:** Association between patient characteristics and type of axillary evaluation in mastectomy patients

Variable		ALND	SLNB	*p*-Value
Year of Diagnosis	1998	557(67.3%)	271(32.7%)	<0.01
	1999	508(61.9%)	313(38.1%)	
	2000	442(52.7%)	396(47.3%)	
	2001	416(46.4%)	480(53.6%)	
	2002	374(41.8%)	521(58.2%)	
	2003	361(31.5%)	785(68.5%)	
	2004	356(29.6%)	847(70.4%)	
	2005	405(30.1%)	940(69.9%)	
	2006	361(23.4%)	1183(76.6%)	
	2007	477(24.6%)	1462(75.4%)	
	2008	775(22.3%)	2696(77.7%)	
	2009	873(21.1%)	3269(78.9%)	
	2010	804(19.4%)	3347(80.6%)	
	2011	746(17.4%)	3540(82.6%)	
Race	Black	1103(35.1%)	2041(64.9%)	<0.01
	White	5978(26.4%)	16704(73.6%)	
	Other	293(21.9%)	1042(78.1%)	
Age	18–46	1784(25.2%)	5305(74.8%)	<0.01
	47–55	1828(24.6%)	5612(75.4%)	
	56–65	1747(26.7%)	4802(73.3%)	
	>65	2096(32.6%)	4331(67.4%)	
Facility Type	Community Cancer Program	710(32.6%)	1465(67.4%)	<0.01
	Comprehensive Community Cancer Program	4527(26.8%)	12346(73.2%)	
	Academic/Research Program	2218(26.2%)	6239(73.8%)	
Facility Location	Midwest	1715(25.4%)	5027(74.6%)	<0.01
	Northeast	1415(26.9%)	3852(73.1%)	
	South	3176(29.8%)	7466(70.2%)	
	West	1149(23.7%)	3705(76.3%)	
Primary Site	Nipple areolar	83(37.2%)	140(62.8%)	<0.01
	Central	474(26.8%)	1292(73.2%)	
	Upper inner	473(25.1%)	1410(74.9%)	
	Lower inner	420(25.1%)	1252(74.9%)	
	Upper outer	2167(28.9%)	5336(71.1%)	
	Lower outer	477(25.6%)	1384(74.4%)	
	Axillary tail	7(22.6%)	24(77.4%)	
	Overlapping lesion	1559(25.5%)	4554(74.5%)	
	NOS	1795(27.8%)	4658(72.2%)	
Tumor size	≤1 cm	1241(25.2%)	3680(74.8%)	0.01
	>1 cm, ≤2 cm	1415(24.2%)	4431(75.8%)	
	>2 cm, ≤3 cm	411(23.3%)	1351(76.7%)	
	>3 cm, ≤4 cm	326(23.7%)	1050(76.3%)	
	>4 cm, ≤5 cm	285(24.2%)	893(75.8%)	
	>5 cm	693(27.4%)	1833(72.6%)	

**Fig. 3:**
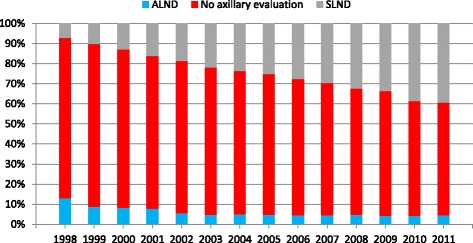
Trend in axillary evaluation over time in patients who underwent breast conservation

The cohort of 55,349 patients who underwent BCT was analyzed separately (Fig. 3). Overall, axillary evaluation increased from 20.1% in 1998 to 43.9% in 2011 (Table 1). Within this group, ALND decreased from 12.9 to 4.5%, while SLNB increased from 7.2 to 39.4%. Similar to TM patients, younger patient undergoing BCT were more likely to have their axilla evaluated than older patients (*p* <0.01). BCT patients were least likely to undergo axillary evaluation at academic/research programs (28.4%) versus community cancer programs (36.4%) and comprehensive community cancer programs (36.6%), (*p* <0.01). Facility locations in the Northeast demonstrated the lowest axillary evaluation rate (27.9%), with the South area reporting the highest rate (41.2%) (*p* <0.01). As with the TM cohort, tumors located near the nipple were the least likely to undergo axillary evaluation (26.7%) versus tumors in the axillary tail (48.1%) (*p* <0.01). Tumors <1 cm were less likely to undergo axillary evaluation (30.3%) than tumors >3 cm (45.3%).

A multivariate analysis of axillary evaluation among BCT patients showed differences by year of diagnosis, facility type and location, as well as patient and tumor characteristics (Table 4). Patients undergoing a surgery procedure in 1999 were the least likely of all years to undergo axillary evaluation, compared with 2011 (OR 0.29 {95% CI 0.24–0.35}). Academic/research programs and facility locations in the Northeast were least likely to evaluate the axilla compared to non-academic cancer centers and centers in the South (OR 0.73 {95% CI 0.69–0.77}) and (OR 0.58 {95% CI 0.55–0.62}), respectively. Tumors <1 cm in size were least likely to undergo axillary evaluation compared with tumors >5 cm (OR 0.50 {95% CI 0.44–0.57}). Black women were 15% less likely to undergo axillary evaluation compared to white women (OR 0.85 {95% CI 0.79–0.92}), as were older women with a 1% decrease in probability of axillary evaluation per year increase in age.

**Table 4:** Multivariate analysis of axillary evaluation and patients’ characteristics among patients who underwent breast conservation

Variable		Odds Ratio	Lower CL	Upper CL	*p*-Value
Age	Per year increase	0.99	0.99	0.99	<0.01
Facility Location (ref = South)	Midwest	0.71	0.67	0.76	<0.01
	Northeast	0.58	0.55	0.62	
	West	0.72	0.67	0.77	
Facility Type (ref = Comprehensive Community Cancer Program)	Community Cancer Program	1.00	0.93	1.08	<0.01
	Academic/Research Program	0.73	0.69	0.77	
Primary Site (ref = Axillary tail)	Nipple areolar	0.39	0.22	0.69	<0.01
	Central	0.54	0.33	0.89	
	Upper inner	0.60	0.37	0.98	
	Lower inner	0.58	0.35	0.94	
	Upper outer	0.69	0.43	1.13	
	Lower outer	0.60	0.37	0.98	
	Axillary tail	0.61	0.373	0.98	
	Overlapping lesion	0.56	0.34	0.91	
Race (ref = White)	Black	0.85	0.79	0.92	<0.01
	Other	0.81	0.73	0.90	
Tumor Size (ref= > 5 cm)	<1 cm	0.50	0.44	0.57	<0.01
	>1 cm, ≤2 cm	0.69	0.71	0.90	
	>2 cm, ≤3 cm	0.89	0.77	1.03	
	>3 cm, ≤4 cm	0.96	0.82	1.13	
	>4 cm, ≤5 cm	0.97	0.81	1.17	
Year of Diagnosis (ref = 2011)	1998	0.30	0.25	0.36	<0.01
	1999	0.29	0.24	0.35	
	2000	0.32	0.27	0.38	
	2001	0.35	0.30	0.41	
	2002	0.37	0.32	0.43	
	2003	0.49	0.43	0.55	
	2004	0.55	0.49	0.62	
	2005	0.56	0.50	0.62	
	2006	0.62	0.56	0.69	
	2007	0.66	0.60	0.73	
	2008	0.77	0.71	0.83	
	2009	0.79	0.73	0.85	
	2010	1.02	0.94	1.10	

## Discussion

The results of this study demonstrate an increased rate of axillary evaluation in DCIS over time, from 44.4% of patients undergoing such a procedure in 1998 to 63.3% in 2011. Despite a favorable trend of increased SLNB (14.9 to 54.2%) with decreased ALND (29.5 to 9.1%) over the time frame of our study, practice patterns nevertheless do not adhere to NCCN and ASCO guidelines regarding axillary evaluation, and demonstrate variation by patient ages, tumor size, geographic location, and practice facility type.

Among patients undergoing BCT in 2011, the most recent year of data capture, the axillary evaluation rate of 43.9% (of which 4.5% is ALND) is concerning. The NCCN and ASCO recommend SLNB only in cases of tumor location precluding future sentinel lymph node mapping, or large volume or mass-associated DCIS [12, 13]. Tumor location that precludes future lymph node sampling is accepted as those tumors located in the central breast, upper outer quadrant, or axillary tail [16]. Overall, these locations accounted for only 15.5% of SLNB in the BCT cohort. Tumors >5 cm in size accounted for only 4.1% of the cohort. Thus, in most of the women in this cohort undergoing BCT, we could not identify clear indication for SLN biopsy.

Though national guidelines recommend SLNB in TM, a significant proportion of patients did not receive any axillary evaluation at all. In terms of patient demographics, those who were not black or were younger in age were less likely to receive axillary evaluation. It is possible that younger patients underwent less morbid staging technique or were perceived as having less aggressive disease than older or black patients. Community cancer programs or facilities located in the western United States were also less likely to evaluate the axilla, which represents regional variation that we saw among all cohorts. Finally, patients with smaller tumors or those located near the nipple/areolar complex understandably underwent the least frequent axillary evaluation, reflecting a potential surgical provider bias that these tumors would be least likely to demonstrate nodal involvement.

While national guidelines recommend SLNB for TM, the ALND rate of 16.3% in our cohort warrants further investigation. Our subgroup analysis of SLNB vs. ALND in TM demonstrated important differences from the overall axillary evaluation group. The total cohort and sub-groups both did show decreased rates of ALND over time. However, our finding that age >65 had the highest rate of ALND is concerning, given the known co-morbidities associated with this procedure and the decreased likelihood of aggressive disease in this age distribution compared with younger women. The subgroup analysis showed that community programs were more likely to utilize ALND than SLNB, potentially reflecting the slower adoption of minimally invasive axillary staging in this setting. The highest rate of ALND in relationship to tumor site and size were in the nipple/areolar region and in tumors >5 cm. This may have reflected failure of intraoperative mapping and/or ALND completed due to high clinical suspicion. Unfortunately, our data are unable to capture this decision-making process.

Other factors demonstrating significant relationship to guideline discordant axillary evaluation in the TM and BCT cohorts included facility type and location. Academic programs were most likely to demonstrate compliance with national guidelines, whereas facility locations in the south were least likely to be compliant. Geographic variation may reflect regional differences in practices that we are unable to capture in our data, but do suggest that a renewed emphasis on adherence with national guidelines, rather than regional practice patterns in the surgical care of DCIS is required.

Our results demonstrate both similarities and differences when compared with other recent studies investigating axillary evaluation in DCIS. Though our rates of BCT for DCIS are similar to other recent studies, we demonstrate higher rates of axillary evaluation in DCIS (63.3%). This may be reflective of our larger and more inclusive NCDB data set. Using the Perspective database, Coromilas et al., showed a 29.2% rate of axillary evaluation [11]. This database primarily captures patients treated at urban and teaching hospitals and has a preponderance of facilities located in the Southern United States. In contrast, the NCDB, with its national catchment area, provides broader geographic representation and also includes smaller community practices and as such is likely to be much more representative of the national practice. Porembka et al. studied the 1998–2002 SEER database and showed a 28% rate of axillary evaluation; however, their study (similar to Coromilas et al.), represented a much smaller population since SEER captures fewer cancer patients than NCDB, and therefore again SEER data may not reflect the trends we observed in a larger, more nationally representative group [10].

Worni et al. utilized the SEER database to study trends in treatment patterns for DCIS, focusing on disease free survival and overall survival [7]. However, they did not highlight whether treatment patterns were compliant with national guidelines, nor detail the variables associated with use of axillary staging. Given the potential to over treat DCIS patients with unwarranted axillary surgery, and the long term complications associated with axillary intervention, understanding factors impacting guideline discordant care is of importance in order to develop strategies for education and improvement in standardization of surgical approaches to DCIS.

Miller et al. recently reported on DCIS and axillary evaluation using the NCDB database, looking specifically at which factors were predictive of tumor upstaging and the relationship to appropriate utilization of SLNB [8]. While our study population has overlap with the recent paper by Miller et al., in order to best capture compliance with axillary surgery for DCIS patients, we excluded from our analysis the subset of DCIS patients upgraded to invasive cancer, for whom SLNB is concordant with care guidelines. Similarly, a study by Nicholson et al., described practice patterns for DCIS care in the United Kingdom and again provided information on axillary staging, but also included patients upgraded to invasive cancer [9]. While these studies collectively provide insight on practice patterns, including axillary interventions, in DCIS patients, our study, focused on the subset with pure DCIS without upgrade, is best positioned to address the question of compliance with axillary staging and hence rates of overtreatment of DCIS patients. We emphasize that our findings provide a benchmark for current practice patterns regarding DCIS and axillary evaluation in the United States, and offer opportunity for improvement in compliance with guidelines.

### Limitations

There are several limitations of our paper. It is a retrospective review that allows limited analysis of pathologic subtypes of DCIS that may warrant more aggressive surgical intervention. We have no data regarding clinical exam or clinical intraoperative judgment that may have led providers to undertake axillary evaluation discordant with national guidelines. Because the NCDB does not have a reliable variable to distinguish between ALND and SLNB, we assumed that lymph node sampling of >4 nodes represented a formal ALND procedure, in line with other similar studies and based on data demonstrating that the majority of women will not have more than 4 SLN [17, 18]. This methodology to distinguish between SLNB and ALND likely resulted in some over-representation of the number of ALND procedures performed. We emphasize this is a significant limitation of our data, though we believe this bias should be small since it is uncommon for greater than four sentinel lymph nodes to exist.

## Conclusions

We report significant rates of non-compliance with national guidelines in the surgical treatment of DCIS, both in the appropriate performance of axillary evaluation and in use of appropriate technique (ALND versus SLNB). The results of this study suggest that increased emphasis be placed on adherence to national guidelines among different facility practice locations and types.

## Abbreviations

ALND: Axillary lymph node dissection
ASCO: American society of clinical oncology
BCT: Breast conservation therapy
DCIS: Ductal carcinoma *in situ*
NCCN: National comprehensive cancer network
NCDB: National cancer database
SLNB: Sentinel lymph node biopsy
TM: Total mastectomy

## References

[1] Kuerer HM, Albarracin CT, Yang WT (2009). Ductal carcinoma *in situ*: state of the science and roadmap to advance the field. J Clin Oncol.

[2] Siegel R, Naishadham D, Jemal A (2013). Cancer statistics, 2013. Ca-Cancer J Clin.

[3] Virnig BA, Tuttle TM, Shamliyan T, Kane RL (2010). Ductal carcinoma *In situ* of the breast: a systematic review of incidence, treatment, and outcomes. J Natl Cancer I.

[4] Ernster VL, Ballard-Barbash R, Barlow WE (2002). Detection of ductal carcinoma *in situ* in women undergoing screening mammography. J Natl Cancer I.

[5] Narod SA, Iqbal J, Giannakeas V, Sopik V, Sun P (2015). Breast cancer mortality after a diagnosis of ductal carcinoma *In situ*. JAMA Oncol.

[6] Liu CQ, Guo Y, Shi JY, Sheng Y (2009). Late morbidity associated with a tumour-negative sentinel lymph node biopsy in primary breast cancer patients: a systematic review. Eur J Cancer.

[7] Worni M, Akushevich I, Greenup R (2015). Trends in treatment patterns and outcomes for ductal carcinoma *In situ*. J Natl Cancer Inst.

[8] Miller ME, Kyrillos A, Yao K (2016). Utilization of axillary surgery for patients with ductal carcinoma *In situ*: a report from the national cancer data base. Ann Surg Oncol.

[9] Nicholson S, Hanby A, Clements K (2015). Variations in the management of the axilla in screen-detected ductal carcinoma *in situ*: evidence from the UK NHS breast screening programme audit of screen detected DCIS. Eur J Surg Oncol.

[10] Porembka MR, Abraham RL, Sefko JA, Deshpande AD, Jeffe DB, Margenthaler JA (2008). Factors associated with lymph node assessment in ductal carcinoma *in situ*: analysis of 1988–2002 seer data. Ann Surg Oncol.

[11] Coromilas EJ, Wright JD, Huang Y (2015). The influence of hospital and surgeon factors on the prevalence of axillary lymph node evaluation in ductal carcinoma *In situ*. JAMA Oncol.

[12] National Comprehensive Cancer Network, Inc. 2015. Version2.2015, 03/11/2015.

[13] Lyman GH, Temin S, Edge SB (2014). Sentinel lymph node biopsy for patients with early-stage breast cancer: American Society of Clinical Oncology clinical practice guideline update. J Clin Oncol.

[14] Woolson RF, Clarke WR. Statistical methods for the analysis of biomedical data. New York: John Wiley & Sons; 2011.

[15] Hosmer Jr DW, Lemeshow S. Applied logistic regression. New York: John Wiley & Sons; 2004.

[16] Kawase K, Gayed IW, Hunt KK (2006). Use of lymphoscintigraphy defines lymphatic drainage patterns before sentinel lymph node biopsy for breast cancer. J Am Coll Surg.

[17] Fleissig A, Fallowfield LJ, Langridge CI (2006). Post-operative arm morbidity and quality of life. Results of the ALMANAC randomised trial comparing sentinel node biopsy with standard axillary treatment in the management of patients with early breast cancer. Breast Cancer Res Treat.

[18] Yi M, Meric-Bernstam F, Ross MI (2008). How many sentinel lymph nodes are enough during sentinel lymph node dissection for breast cancer?. Cancer.

